# Evaluation of the Treatment Process of Landfill Leachate Using the Toxicity Assessment Method

**DOI:** 10.3390/ijerph13121262

**Published:** 2016-12-21

**Authors:** Aifeng Qiu, Qiang Cai, Yuan Zhao, Yingqing Guo, Liqian Zhao

**Affiliations:** 1 School of Environmental and Safety Engineering, Changzhou University, No. 1 GeHu Road, Wujin District, Changzhou 213164, Jiangsu, China; captainqiu1013@163.com (A.Q.); zhaoyuan12@tsinghua.org.cn (Y.Z.); water8820@163.com (Y.G.); 2 Yangtze Delta Region Institute of Tsinghua University, No. 705, Yatai Road, Nanhu District, Jiaxing 314006, Zhejiang, China; liqian_zhao@hotmail.com

**Keywords:** landfill leachate, electrolytic biological filter, *Vibrio fischeri*, zebrafish larvae, zebrafish embryos, toxicity reduction

## Abstract

Landfill leachate is composed of a complex composition with strong biological toxicity. The combined treatment process of coagulation and sedimentation, anaerobics, electrolysis, and aerobics was set up to treat landfill leachate. This paper explores the effect of different operational parameters of coagulation and sedimentation tanks and electrolytic cells, while investigating the combined process for the removal efficiency of physicochemical indices after processing the landfill leachate. Meanwhile, a battery of toxicity tests with *Vibrio fischeri*, zebrafish larvae, and embryos were conducted to evaluate acute toxicity and calculated the toxicity reduction efficiency after each treatment process. The combined treatment process resulted in a 100% removal efficiency of Cu, Cd and Zn, and a 93.50% and an 87.44% removal efficiency of Ni and Cr, respectively. The overall removal efficiency of chemical oxygen demand (COD), ammonium nitrogen (NH_4_^+^-N), and total nitrogen (TN) were 93.57%, 97.46% and 73.60%, respectively. In addition, toxicity test results showed that the acute toxicity of landfill leachate had also been reduced significantly: toxicity units (TU) decreased from 84.75 to 12.00 for zebrafish larvae, from 82.64 to 10.55 for zebrafish embryos, and from 3.41 to 0.63 for *Vibrio fischeri*. The combined treatment process was proved to be an efficient treatment method to remove heavy metals, COD, NH_4_^+^-N, and acute bio-toxicity of landfill leachate.

## 1. Introduction

Landfilling is still a widely employed method for municipal waste treatment [1]. A landfill operates like a massive anaerobic bioreactor which contains physically, chemically, and biologically heterogeneous systems [2]. Leachate is generated from landfill, which is caused by the percolation of rainwater and moisture mixed with the degradation of solid waste [3]. Landfill leachate is a sort of organic refractory effluent with complex pollutant constituents and high concentrations of poisons [4,5], including high contents of organic matter (biodegradable and biorefractory), inorganic substances (metals, NH_4_^+^-N, and chlorides), and xenobiotics consisting of aromatics, phthalates, phenols, pesticides, and other pollutants [6,7]. It is extremely difficult to treat and may impact environmental quality [8], groundwater, and human health [9,10] without improper treatment process [11].

In order to minimize the toxicity of landfill leachate, several treatment methods have been applied to landfill leachate, including advanced oxidation technology, air stripping methods, adsorption methods, membrane treatment methods, etc. [2]. Advanced oxidation processes have proven to be an efficient method to degrade refractory organic pollutants, including ozone oxidation, photocatalytic oxidation, the Fenton method, the electrochemical oxidation method, and so on [12]. Chemical oxygen demand (COD) and NH_4_^+^-N removal efficiency of landfill leachate were 90.3% and 80.1%, when treated by electrochemical oxidation combined with adsorption pretreatment [13]. An effective process was achieved when applying titanium coated with lead dioxide (PbO_2_) or tin dioxide (SnO_2_) as the anode, leading to decolorized leachate, COD reduced to 100 mg/L, and 100% removal of NH_4_^+^-N [14]. The electrochemical oxidation method is commonly utilized for its robustness, versatility, and decreased amount of sludge, easy operation with little or no chemical additions [15,16], which could effectively degrade complex organic compounds and improve the biodegradability of recalcitrant contaminants [17]. The process is based on effluent electrolysis, including the oxidation of pollutants in the electrolytic tank, which consists of two electrodes connected by an external circuit to ensure the electrochemical oxidation reaction [18,19]. In this paper, the combined treatment process can tolerate more shock load of landfill leachate and have more processing functions than single conventional biological method. The coagulation and sedimentation process can remove suspended materials, and color to improve the biodegradability. The anaerobic is mainly used for further removal of heavy metals and organic compounds, reducing the concentration of pollutants to shorten the time of the electrochemical oxidation process. The electrochemical oxidation process can effectively degrade organic compounds, which can improve the biodegradability and create conditions for the next step of the aerobic reaction. The aerobic process focuses on the further removal of COD and NH_4_^+^-N.

Considering there were a lot of non-degraded organics, evaluation of toxicity was necessary, especially for the electrochemical oxidation process, which promises a decrease of toxicity. Meanwhile, landfill leachate effluent has reached the water discharge standard according to the relevant legislation after treatment, and the interactions of its chemicals and toxic degradation products with the biota occurred as well [8]. The effluent ecotoxicity cannot be reduced even though color, COD and total organic carbon (TOC) have been significantly reduced [20,21]. The environmental safety risk of raw or treated landfill leachate usually evaluated refers to the analysis results of the regular physicochemical indices of landfill leachate. Therefore, it was necessary to conduct a bioassay before discharging into a natural recipient in order to reduce the risk of biological effects [4]. Acute toxicity was frequently used in organisms exposed in effluent to reflect the ecotoxicity [22,23]. Model organisms, such as bacteria, algae, plants, invertebrates, and fish have been used to assess landfill leachate toxicity [24]. The toxicity assessment methods of landfill leachate are mature, but it has only been used to evaluate the effluent. Studies have reported that the mortality rate of zebrafish was used to evaluate the reduction of acute toxicity in dye effluent treated by the Fenton-coagulation process [20]. The *Brachydanio rerio* and *Poecilia vivipara* showed that the toxicity in leachate had hardly been reduced by Ozonation [25]. If it could evaluate the treatment process, it may improve the efficiency of the process for toxicity reduction. In this paper, a series of bioassays with different organisms were used to evaluate the bio-toxicity of different process effluents, such as *Vibrio fischeri*, zebrafish embryos, and zebrafish larvae. The zebrafish embryos were sensitive indicators of toxic pollutants in industrial and landfill leachate, and zebrafish larvae were also applied to evaluate toxic components independently [26,27]. This paper is mainly aimed at evaluating the effectiveness of the combined treatment process with respect to the removal of both pollutants and acute toxicity. The goal was to: (i) explore the influence of operating conditions, with the main focus on the coagulation and sedimentation, and electrolysis processes; and (ii) calculate the toxicity reduction efficiency of different processes to evaluate the combined treatment process.

## 2. Materials and Methods

### 2.1. Sample

Four hundred liters of landfill leachate were collected from the waste landfill site in Jiaxing City during March 2016 (Zhejiang Province, China).The raw sample was mixed with new and old leachate in the production process of landfill site. The content of TN, and NH_4_^+^-N were higher, the concentration of total phosphorus was low, and so the proportion of nutrients was seriously unbalanced. In addition, the water also contained many types of heavy metals.

### 2.2. Physicochemical Tests

The physicochemical parameters of the raw samples and four different treatment process effluents were measured by the following methods. Values of each parameter tested were mean ± SD from five different measurements. The COD was tested by the dichromate method (GB/T11914-1989, in Chinese); NH_4_^+^-N was determined by the Nessler’s reagent spectrophotometry (GB/T7479-1987, in Chinese); the determination of total phosphorus (TP) was the Ammonium molybdate spectrophotometric method (GBT11893-1989, in Chinese); the TN was measured by the alkaline potassium persulphate digestion-UV spectrophotometric method (HJ 636-2012, in Chinese); a pH meter was used to evaluate the pH level (GB/T6920-1986, in Chinese). Heavy metals (Cd, Cr, Pb, Ni, Zn and Cu) were determined by Atomic Absorption Spectrophotometry (240 Duo (Zeeman & FS), Agilent, Santa Clara, CA, USA) and determination of Cl^−^ by Ion Chromatography (CIC-300, SHINEHA, Qingdao, China).

### 2.3. Experimental Device

**Figure 1:**
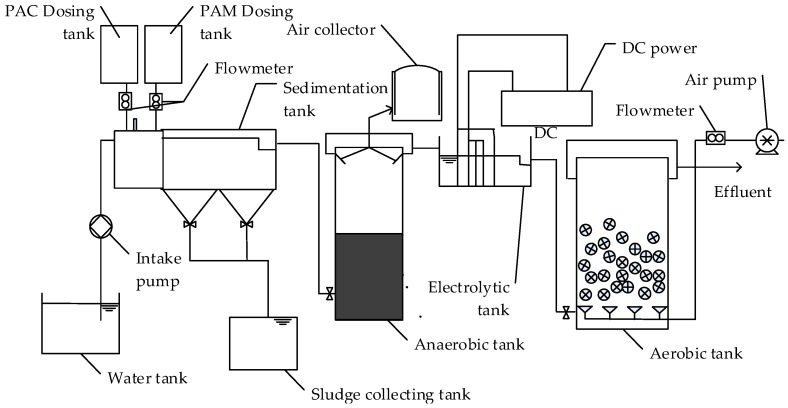
Experimental device for the treatment process.

The combined electrochemical oxidation reactor structure is shown in Figure 1 and the experiment was divided into four stages, including coagulation and sedimentation, anaerobic reaction, electrochemical oxidation, and aerobic reaction. All of the tanks in the process were made of organic glass.

In the coagulation and sedimentation stage, a 2 L poly aluminum chloride (PAC) dosing tank, a 2 L polyacrylamide (PAM) dosing tank, a 2 L coagulation tank and a 4 L sedimentation tank were connected with plastic pipes. A flowmeter was applied to control the flow of added medicament. The sedimentation tank was connected with a sludge collecting tank. The 48 L anaerobic tank was the main reactor in the anaerobic reaction stage, which was connected with a gas collector for releasing methane gas. A built-in heating rod was installed to control the reaction temperature. Then the water sample was electrochemically oxidized in the 4 L electrolytic cell, in which three Ru/Ir/TiO_2_ coated cathodes and two stainless steel anodes was installed. All of these electrodes, connected to a DC power source, were planar with a 100 mm × 100 mm contact surface, and spaced 10 mm apart. A baffle was arranged on the water inlet of the electrolytic cell to prevent the water flow from drying up. The fourth stage was the aerobic reaction, and the effective volume of the aerobic tank was 51 L. The process adopted a bottom in-flow, bottom aeration, and overflow of water, and used a self-developed filler with a diameter of 25 mm.

### 2.4. Toxicity Tests

Water samples for ecotoxicity tests were from raw leachate and the four treatment process effluents. Samples were designed as follows: Raw was the raw landfill leachate, sample A was collected from the coagulation and sedimentation tank effluent, sample B was from the anaerobic tank effluent, sample C was from the electrochemical oxidation tank effluent, and sample D was from the aerobic tank effluent. All of these samples were stored at 4 °C. Toxicity tests were performed within 36 h after sample collection [28].

#### 2.4.1. Zebrafish Toxicity Test

All adult zebrafish (AB strain) were approved by the laboratory to establish a long-term stable culture and mature zebrafish. The control conditions were as follows: the light period followed a 14 h: 10 h light: dark cycle; temperature was controlled at 28 ± 0.5 °C; hardness, 250 mg/L; pH, 7.5 ± 0.5; dissolved oxygen, 10.5 ± 0.5 mg/L. The zebrafish were fed twice each day with freshly hatched shrimp eggs and once daily with flake food (Tetra, Melle, Germany). The dilution buffer was KCl, 0.006 g/L, MgSO_4_·7H_2_O, 0.123 g/L, NaHCO_3_, 0.065 g/L, and CaCl_2_·2H_2_O, 0.294 g/L. Before the collection of zebrafish eggs, the well-fed adult males and females with a ratio of 2:1 (separated by a divider) were put into breeding boxes containing grids at the bottom to keep the eggs from being eaten. Spawning was induced by the light irritation of the next morning (starting within 30 min). The eggs were washed several times by Hanks solution and incubated in embryo media. Before experimentation, the eggs were observed under a microscope (TS100-F, Nikon, Tokyo, Japan) and normal ones were maintained in culture dishes loaded with Hanks solution.

The zebrafish larvae 96 h acute toxicity test was implemented by the procedure described in ISO 7346-1 1996 [29]. As shown in Table 1, different samples were diluted to five concentrations with standard buffer to adjust the sensitivity region. In addition, the bioassay was carried out in the same conditions as stock cultures, triplicated for each concentration and one for blank control. Ten healthy developed larvae of *D. rerio* (at approximately 120 hpf) were transferred to each culture dish with 15 mL dilutions of samples. The dead organisms were counted and removed at 24 h, 48 h, 72 h, and 96 h after exposure to the samples. The acute toxicity was characterized by the 96 h median lethal concentration (LC_50_) values.

The zebrafish embryo experiment was also applied to those samples. The experimental concentration was set as shown in Table 1. Exposure experiments were carried out under the same conditions as the stock culture. Fertilized embryos (at approximately 3 hpf) kept in 24-well cell culture plates with one normal embryo exposed to 2 mL of test solution per well, a total of 20 embryos for per test sample, and the last four wells remained as to be controls with the Hanks solution. The experiment was conducted in triplicate for each concentration sample and the control groups were used. For all groups, the early embryonic development was observed, as were mortality, hatching, and malformation by a microscope at each interval of 24 h, with the removal of dead embryos. During the 72 h exposure experiments, an account of each group for mortality, hatching rate, and malformation rate was kept [30]. After exposure, the 72 h LC_50_ (EC_50_) values were used to evaluate the acute toxicity.

#### 2.4.2. Luminous Bacteria Toxicity Test

For the acute toxicity test, *Vibrio fischeri* powder (purchased from J and Q Environmental Technologies Co., Ltd., Beijing, China) was recovered using 3% NaCl solution at 20 °C for 15 min. All samples for bioassay were diluted using 3% NaCl solution with, respectively, 100%, 50%, 25%, 12.5%, 6.25%, and 3.125% dilution degrees. After 15 min exposure to the samples in the 96-wells microplate, the relative luminescence intensity was determined by a GloMax-Multi Detection System (Promega Co., Madison, WI, USA). Samples were tested in triplicate and one for blank control. Toxicity was evaluated by the inhibition ratio, which was calculated by the following equation. LU was the relative light unit (RLU) of *Vibrio fischeri* exposed to the experimental group, and LU_0_ was the RLU to the blank control [31].

(1) $$X\left(\%\right)=\left(1-\frac{\mathrm{LU}}{{\mathrm{LU}}_{0}}\right)\times 100\%$$

#### 2.4.3. Methods of Toxicity Evaluation

TU was used to evaluate the toxicity of collected water samples, which was calculated based on the following formula:

(2) $$\mathrm{TU}=\frac{100\%}{{\mathrm{LC}}_{50}\left({\mathrm{EC}}_{50}\right)}$$

However, due to limitation of sensitivity of the biomarker, the organisms, exposed to samples with low toxicity, the inhibition rate was less than 50%, so TU was calculated by the following formula:

(3) TU=RE×0.02×100

where RE represents the mortality of zebrafish larvae and embryos, and the relative inhibition intensity of *Vibrio fischeri* after being exposed to samples.

Statistical analysis was performed using Origin 8.5 (Origin Lab., Northampton, MA, USA). Calculated samples of the median lethal concentration (LC_50_) and the median effect concentration (EC_50_) on *D. rerio* and luminous bacteria. Data were shown as the mean and standard error and evaluated by using one-way analysis of variance (ANOVA). The exposure group is significantly different from the control group (*p* < 0.05).

## 3. Results

Physicochemical indices of the raw samples are shown in Table 2.

### 3.1. Coagulation and Sedimentation Process

The amount of water was 1.5 L/h, the hydraulic retention time was 4 h, and the stirring speed was set to 100 rpm. Leachate needs to be coagulated with PAC and PAM as coagulant aids in the experiment: the effective content of PAC was 72%. PAM was a cation with a molecular weight of 10 million. The pH value of the landfill leachate was 6.55. Under acidic conditions, PAC would produce a positively-charged hydrolyzate while the landfill leachate contains large amounts of negatively-charged humic acid, and thus gene efficiency electrical neutralization reaction to form aluminum humates and Settlement [32].

The optimum operational conditions to remove pollutants for this process have been obtained in previous studies. Table 3 shown the main characteristics of landfill leachate through the treatment process. Concerning the removal efficiency of COD and economic factors, the most efficient conditions of using PAC as a coagulant and PAM as a coagulant aid are: dosage of PAC and PAM at 1.2 g/L, and 1 mg/L, respectively. The removal efficiency of COD was 56.64%. The effect of removing NH_4_^+^-N was not obvious, at about 13.71%. The removal efficiency of Zn, Cd, Ni, Cr, and Cu were about 98.32%, 82.56%, 4.46%, 68.80%, and 100%, respectively.

**Figure 2:**
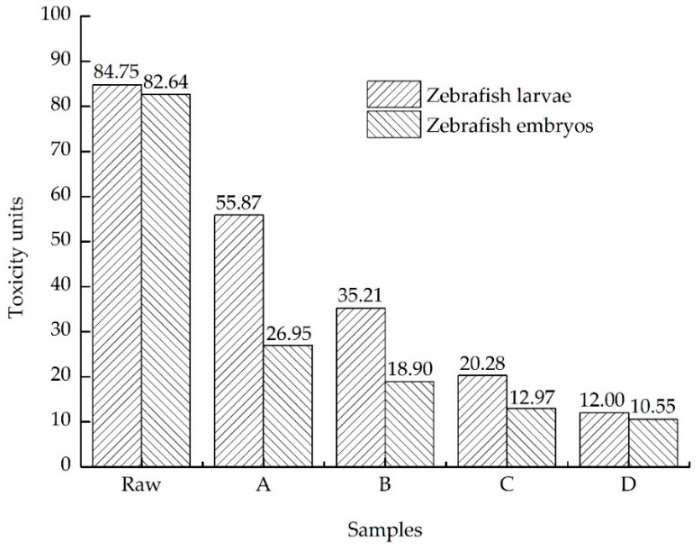
Toxicity units of different samples by zebrafish larvae and zebrafish embryos. Raw, the raw landfill leachate; sample A, coagulation and sedimentation tank effluent; sample B, anaerobic tank effluent; sample C, electrochemical oxidation tank effluent; and sample D, aerobic tank effluent.

**Figure 3:**
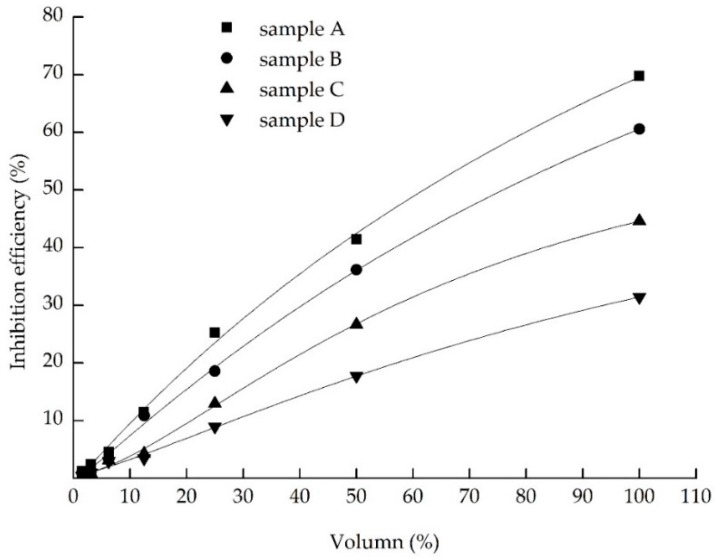
Dose-response relationship of four treatment process effluents on the Vibrio fischeri toxicity. sample A, coagulation and sedimentation tank effluent; sample B, anaerobic tank effluent; sample C, electrochemical oxidation tank effluent; and sample D, aerobic tank effluent.

To clarify the toxicity reduction efficiency of effluent after the coagulation and sedimentation process, it is necessary to consider the evolution of acute toxicity. As shown in Figure 2 and Figure 3, 96 h LC_50_ of the effluent was about 1.79% and the TU value was 55.87 according to zebrafish larvae, 72 h LC_50_ of the effluent was about 3.71% and the TU value was 26.95 according to zebrafish embryos. The measured 15 min EC_50_ of the effluent was about 61.86% and the TU value was 1.62 according to *Vibrio fischeri*. Toxicity of the landfill leachate had been reduced significantly after the process, which led to the reduction of toxicity in zebrafish larvae by 34.08%, in zebrafish embryos by 67.39%, and to *Vibrio fischeri* by 52.49%, according to the value of TU.

### 3.2. Anaerobic Process (Sludge Acclimation and Up-Flow Anaerobic Sludge Bed (UASB) Start)

Effluent from the coagulation and sedimentation tank went through the pipe into the bottom of the UASB. The hydraulic retention time was 32 h. Startups of UASB after 25 days’ acclimation of granular sludge, UASB ran for 5–15 days, COD removal efficiency fluctuated significantly, and the removal efficiency of COD was stable at 8%–10% after 15 days. A month after the start of the reactor, the index tends to stabilize, with the successful startup of the UASB reactor. The index of the effluent was measured in Table 3. The COD removal efficiency of the anaerobic effluent was no more than 10%, which was probably due to complex organic matters, like the poorly biodegradable and non-biodegradable organic substances in landfill leachate. The removal efficiency of Zn, Cd, Ni, and Cr were 89.80%, 100%, 52%, and 31.17%, respectively.

In the anaerobic process, the 96 h LC_50_ value of effluent was calculated at about 2.84%, nearly equal to the TU value of 35.21 for zebrafish larvae. The 72 h LC_50_ value was 5.29%, nearly equal to the TU value of 18.90 for zebrafish embryos, and 15 min EC_50_ value was 75.88%, nearly equal to the TU value of 1.32 for *Vibrio fischeri*. The toxicity decreased by 36.98% for zebrafish larvae, by 29.87% for zebrafish embryos, and by 18.52% for *Vibrio fischeri*, which was comparable to the coagulation and sedimentation process.

### 3.3. Electrochemical Oxidation Process

This stage investigates the effect of current density and pH on electrolysis. The electrochemical oxidation process was divided into direct and indirect oxidation during the treatment of landfill leachate. The direct oxidation method uses anode surface to strengthen the ·OH oxidation reaction with organic matter. Indirect oxidation is mainly used for the removal of organics and NH_4_^+^-N in the leachate [12]. The main reaction formulae are as follows:

2Cl^−^ → Cl_2_ + 2e^−^ (4)

Cl_2_ + H_2_O → HClO + Cl^−^ + H^+^ (5)

HClO → H^+^ + ClO^−^ (6)

ClO^−^ + organics → CO_2_ + H_2_O + Cl^−^ (7)

HOCl + NH_4_^+^ → NH_2_Cl + H_2_O + H^+^ (8)

HOCl + NH_2_Cl → NHCl_2_ + H_2_O (9)

NHCl_2_ + H_2_O → NOH + 2H^+^ + 2Cl^−^ (10)

NHCl_2_ + NOH → N_2_ + HOCl + H^+^ + Cl^−^ (11)

The Cl^−^ at the anode surface is oxidized to Cl_2_, and Cl_2_ was dissolved in water to produce a strong oxidizing HClO to decompose organic matter and NH_4_^+^-N. The main factors that affect the electrolytic effect include pH, current density, electrolytic time, and concentration of Cl^−^, etc. [33,34]. The concentration of Cl^−^ in different pH conditions, the amount of available chlorine, like ClO^−^, is different, which affects the removal efficiency of organic compounds, and so on. The current intensity increases with the increase of the current density, and as chlorine by electrolysis increases, the effect of electrochemical oxidation will improve, but this also increases energy consumption.

The concentration of Cl^−^ was 2200–2400 mg/L after the anaerobic reaction. While using electrochemical oxidation treatment of landfill leachate, adding Cl^−^ in a certain range would increase the efficiency of the electrolytic reaction, which was conducive to the removal of pollutants. However, if the concentration of Cl^−^ was greater than 2000 mg/L, it would increase the risk of organic chlorine and the toxicity of the effluent [35]. Therefore, the concentration of Cl^−^ in the landfill leachate did not need to be adjusted.

#### 3.3.1. Effect of Current Density on Electrochemical Oxidation

**Figure 4:**
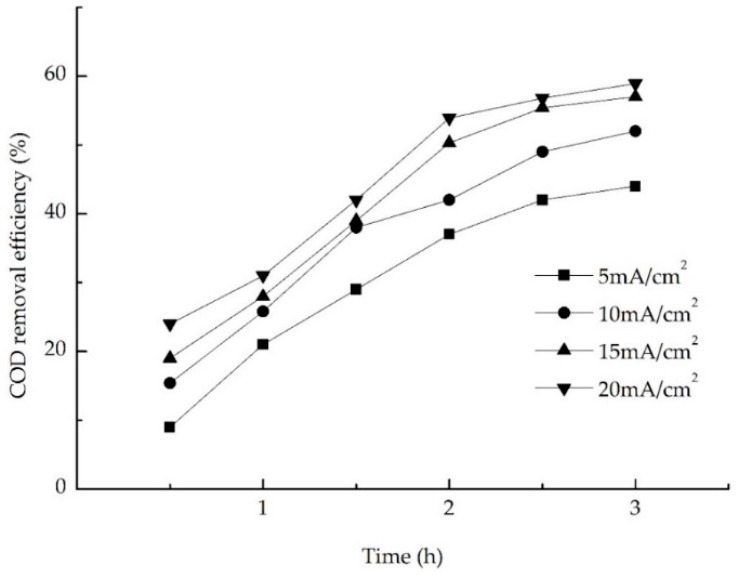
Effect of current density on chemical oxygen demand (COD) removal.

Under the higher current density, the activity of the denitrifying bacteria was affected, which then affected the removal efficiency of NH_4_^+^-N. Considering energy consumption and capacity of the plate, it was very important to find the suitable current density. Figure 4 shows that with an electrolysis time within 3 h, the removal efficiency of COD increases with the increase of the current density, and tends to be steady at 2.5–3 h. With an electrolysis time of 2.5 h, an increase in the current density increased from 5 mA/cm^2^ to 10 mA/cm^2^, 15 mA/cm^2^, and 20 mA/cm^2^ yielded an increase in the efficiency of COD removal from 42.0% to 49.00%, 55.40%, and 56.80%, and it does not change significantly after 15 mA/cm^2^. Taking this test into account, the best current density obtained for the electrolytic tank was 15 mA/cm^2^.

#### 3.3.2. Effect of pH on Electrochemical Oxidation

**Figure 5:**
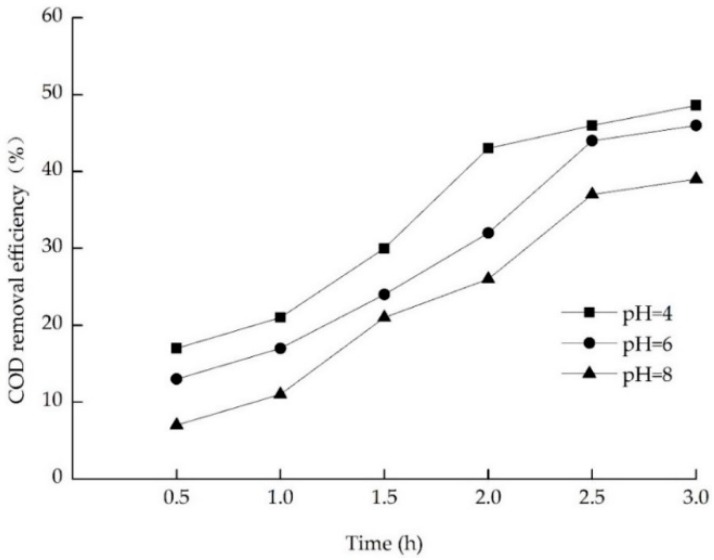
Effect of pH on COD removal.

It has been widely accepted that pH is significant in the electrochemical oxidation process [21]. Research shows that the degradation efficiency of NH_4_^+^-N, COD, current density, and energy consumption were higher in strong acid and strong alkaline conditions than that under weak alkaline conditions [33]. As could be seen in Figure 5, in 0.5–2.5 h, COD removal efficiency of landfill leachate of pH in three kinds showed a rapid growth trend, and tends to be gentle in 2.5–3.0 h. At 3 h, an increase in the pH from 4 to 6 and 8 yielded results in an increase in the efficiency of COD removal from 48.60% to 46%, and 39%, it did not change significantly after a pH of 6. This indicated that a high pH value is not conducive to the removal of COD in landfill leachate. Since the pH value of the influent of the electrolytic tank was about 6, it was not necessary to regulate the raw leachate pH, taking into account the raw leachate pH and cost.

The index of the effluent was measured in Table 3 and the removal efficiency of COD and NH_4_^+^-N were 61.47% and 73.65%, respectively. The process could reduce the toxicity, results in Figure 2 and Figure 3 showed that the mean value of 96 h LC_50_ for the effluent was about 4.93%, nearly equal to the TU value of 20.28 for zebrafish larvae. The 72 h LC_50_ value was 7.71%, nearly equal to the TU value of 12.97 for zebrafish embryos. The inhibition rate of the 100% concentration effluent was lower than 50% for Vibrio fischeri and the TU value was 0.89. Toxicity decreased by 42.40% for zebrafish larvae, by 31.38% for zebrafish embryos, and by 32.58% for *Vibrio fischeri* in the electrochemical oxidation process.

### 3.4. Aerobic Process

In this stage, the hydraulic retention time was 34 h. Inoculation of biofilm was used as the hang bio-membrane method of the aerobic tank. Glucose was proved to be the optimal carbon source, ammonium chloride was chosen as the nitrogen source, dipotassium hydrogen phosphate as phosphorus source, and it was confirmed that the C:N:P in the water body was 100:5:1. Table 3 showed that the pollutants in the wastewater were not entirely eliminated by aerobic reaction. A nitration reaction occurred under aerobic conditions, and treatment of the ammonia of the landfill leachate oxidation to nitrate and nitrite by aerobic denitrifying bacteria reduced the concentration of NH_4_^+^-N.

In previous studies, COD and NH_4_^+^-N concentration in the effluent were 90 mg/L and 10 mg/L respectively, which shows the membrane formed successfully. The removal efficiency of COD, NH_4_^+^-N, Cr, and Ni in the aerobic reactor tends to be stable for about 59.09%, 87.95%, 22.50%, and 25%, respectively after running 10–15 days. The 96 h LC_50_ of zebrafish larvae exposed to effluent was 8.33%, nearly equal to the TU value of 12.00. The 72 h LC_50_ value was 9.48%, nearly equal to the TU value of 10.55 for zebrafish embryos, and the TU value was 0.63 for *Vibrio fischeri*. Toxicity decreased by 40.83% for zebrafish larvae, by 18.66% for zebrafish embryos, and by 29.21% for *Vibrio fischeri* in the aerobic process.

### 3.5. Combined Landfill Leachate Treatment

The effluent indices could reach the standards for pollution control on the landfill site for domestic waste (GB16889-2008) after the treatment of combined processes. As a whole, it was observed that the removal efficiency of COD, NH_4_^+^-N, and TN was 93.57%, 97.46%, and 73.60%, respectively. At the same time, five heavy metals in the landfill leachate were remarkable with 100% removals of Cu, Cd, and Zn, while the removal efficiency of Ni and Cr was 93.50% and 87.44%. Consequently, the results showed that the combination of biological and chemical technology could effectively remove the heavy metals (Cu, Zn, Cd, Cr, and Ni), NH_4_^+^-N, and COD in the landfill leachate.

**Figure 6:**
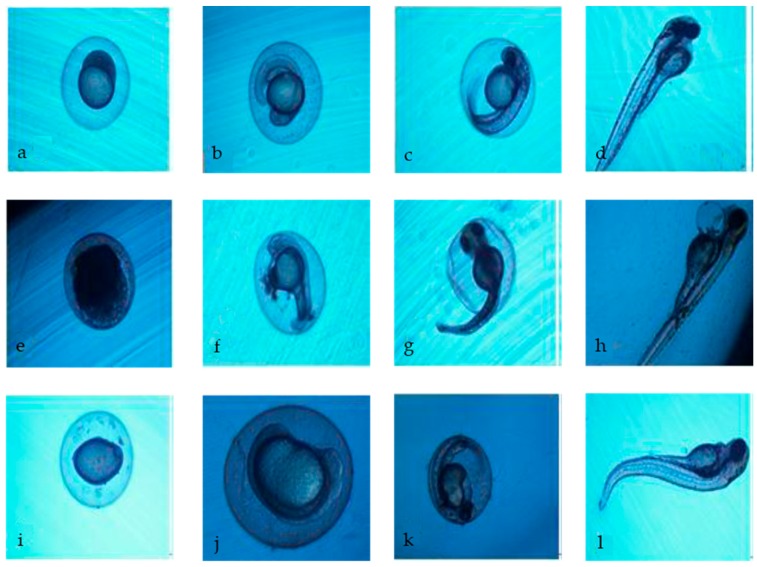
Effect on the early development of zebrafish embryos exposed to diluted landfill leachate. (**a**–**d**) normal development; (**e**) 6 h-coagulated egg; (**i**) 12 h-egg growing block; (**f**) 24 h-pericardial bleeding; (**j**) 24 h-no extension of the embryonic tail; (**g**) 48 h-deformities of spine; (**k**) 48 h-abnormal yolk and pigmentation reduction; (**h**) 72 h-pericardial edema; (**l**) 72 h-deformities of the tail.

An appreciable decrease of toxicity was achieved by the combined treatment process, the toxicity of landfill leachate was reduced by 85.83%, which was evaluated by 96 h acute toxicity to zebrafish larvae, an 87.29% toxicity reduction of 72 h acute toxicity to zebrafish embryos, and an 81.52% toxicity reduction to *Vibrio fischeri.* In 72 h acute toxicity to zebrafish embryos, the EC_50_ value of mortality, hatching, and malformation of raw landfill leachate were 1.21%, 1.12%, and 1.33%, respectively. As shown in Table 4, the 72 h EC_50_ value of hatching rates of zebrafish embryos exposed to the four treatment process effluents were 3.66%, 5.25%, 7.49%, and 8.95%, respectively. Additionally, statistical analysis suggests that the hatching rate has significantly increased with further treatment. The 72 h EC_50_ value of the coagulation and sedimentation tank, and the anaerobic tank effluents for the zebrafish embryos’ malformation were 4.68% and 5.41%. As show in Figure 6, the raw landfill leachate and effluent from the coagulation and sedimentation tank and anaerobic tank in the high concentration (in the case of non-lethality) will lead to more than four kinds of toxicity indicators occurring in zebrafish embryos simultaneously, for instance, growing block, pigmentation reduction, pericardial edema, and associated with bradycardia, deformities of spine and tail, abnormal yolk sac, etc. In addition, exposure to lower mass concentrations (less than half the lethal mass) of samples only produce one or two toxicity indicators simultaneously than those mentioned above, which are usually shown as deformities of the spine and tail.

## 4. Discussion

### 4.1. Reduction of Physicochemical Characters and Toxicity in the Landfill Leachate

The coagulation and sedimentation process was simple and easy to operate, and not affected by the change of water quality and quantity [20]. It was observed that the COD was removed by 56.64% in the process, which represents the reduction of toxicity. The removal effect of COD was better than NH_4_^+^-N. The small reduction of NH_4_^+^-N was also observed in other trials [36], due to the electrostatic attraction of ammonium ions to the negatively-charged colloidal particles, which were removed afterwards by the electrochemical oxidation process. The reduction in toxicity was probably due to the removal of heavy metals and toxic organic pollutants from the landfill leachate by the charge neutralization reaction in the coagulation and sedimentation tank. Metals leached from landfill would bond to organic matter [37]. The dark color of landfill leachate is the presence of high concentrations of humic substances which represent the mostly organic compounds [38]. Furthermore, the sedimentation of toxic pollutants could also result in the reduction of the acute toxicity. Among these model organisms, significant detoxification regarding zebrafish embryos was observed in the process.

In the anaerobic process, Studies have shown that NH_4_^+^-N promotes the methane production when the concentration of NH_4_^+^-N was lower than 0.4 g/L [39]. Additionally, organic pollutants in the effluent were decomposed into methane, carbon dioxide and other substances. Moreover, some of the organic compounds were synthesized into bacterial cells and removed by the effects of anaerobic microorganisms, which will reduce the partial toxicity. The final removal efficiency could be derived as the microorganisms in the reactor may have resulted in a certain adsorption of heavy metals, while some heavy metals may be adsorbed by granular sludge and then transferred into sludge. To summarize, the decrease of toxicity was not only due to the removal of the toxic organic compounds from the anaerobic reaction, but also the conversion of the highly toxic organic matter to less material. The toxicity reduction of the anaerobic effluent was related to the removal of heavy metal ions in the wastewater. When heavy metal ions enter the tank, they were combined with bases of enzymes or proteins to cause denaturation and deactivation [25]. Trace amounts of heavy metal ions could accumulate in the cells and eventually produce toxic effects in microorganisms. Other research has shown that the order of toxicity of heavy metal ions, Cr > Cd > Cu > Zn > Ni Cr, showing which elements are the most toxic, and the toxicity of various heavy metal ions showed more than one kind of toxicity. In this part of the process, the removed efficiency of Cr was as low as 33%, leading the toxicity of the anaerobic treatment system. Furthermore, the organic matter in the wastewater which was difficult to be degraded or not biodegradable had a serious impact on anaerobic microorganisms, affecting the reduction of the toxicity of the effluent.

It was the most commonly used electrochemical procedure for removing organic pollutants from wastewaters [19], which could reduce the toxicity of the wastewater. According to previous studies, the toxicity of landfill leachate was mainly caused by the recalcitrant organic fraction. An appropriate reaction time and current density of the electrochemical oxidation treatment for the effluent led to the decrease in toxicity [40]. It is quite conceivable that the reason for organic substance mineralization and partial oxidation was the electrochemical oxidation process, which the current intensity increased more obviously. The greater the current intensity, the higher the oxidation efficiency of organic compounds and the greater the toxicity decreased. Meanwhile, COD has been greatly reduced, which means a large amount of organic compounds and NH_4_^+^-N has been reduced, thereby reducing the toxicity of the effluent.

It could be seen from Table 3 that the removal efficiency of COD and NH_4_^+^-N reached 59.09% and 87.95%, and resulted in a reduction of toxicity by 40.83%. On the one hands, the factors associated with toxicity reduction were hydraulic retention time and sludge age, and the greater the hydraulic retention time, the longer the sludge age, and the more efficient the reduction of toxicity. On the other hand, organic pollutants were adsorbed, condensed, and decomposed by the biological membrane in the aerobic pool, and finally produced the synthetic cell body, as well as carbon dioxide and water, which were the cause of the toxicity reduction.

### 4.2. Analyze the Effect of Different Treatment Process

**Figure 7:**
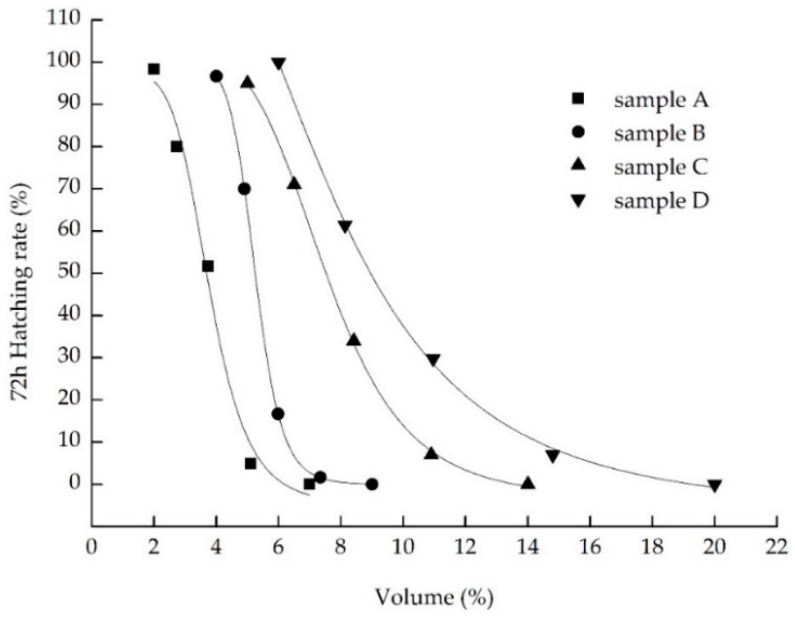
Seventy-two hour hatching rate on the early embryos of zebrafish exposed to four treatment process effluents. Sample A, coagulation and sedimentation tank effluent; sample B, anaerobic tank effluent; sample C, electrochemical oxidation tank effluent; and sample D, aerobic tank effluent.

After a series of treatment processes for the landfill leachate, the final effluent was still toxic for the species used for the evaluation of landfill leachate toxicity, while the toxicity of the final effluent was significant lower than the raw landfill leachate. The EC_50_ value of raw landfill leachate for mortality and hatching rate of zebrafish embryos indicated that landfill leachate caused significant inhibition in zebrafish embryos hatching, and the effluent toxicity test of the other four treatment processes also verified this phenomenon. Combining Figure 7 and the value of EC_50_, the coagulation and sedimentation, and electrochemical oxidation processes have removed the pollutants that inhibit the hatching of embryos. Table 4 shows that the effluent did not produce malformation in zebrafish embryos after the electrochemical oxidation process. In other words, the toxic substances that could cause the malformation of zebrafish embryos were removed by the electrochemical oxidation process.

The study has shown that when zebrafish embryos were exposed to the samples without dilution, they condensed and died quickly. For the toxicity test of zebrafish larvae, the formula of toxicity units that calculated the toxicity reduction efficiency of each treatment process were 34.08%, 36.98%, 42.40% and 40.83%, respectively. The most efficient method for toxicity reduction was the electrochemical oxidation process. For the toxicity test of zebrafish embryos, the toxicity reduction efficiency of each treatment process were 67.39%, 29.87%, 31.38% and 18.66%, respectively. The coagulation and sedimentation process removed the most toxicity from the landfill leachate, which was found effectively to remove large molecular weight molecules [41]. The following describes the electrochemical oxidation process. Moreover, for the toxicity test of *Vibrio fischeri*, as Figure 3 described, the toxicity decreased by 52.49%, 18.52%, 32.58% and 29.21%, respectively and the luminescence inhibition rate of the effluent was less than 50% after the process of electrochemical oxidation, which illustrated that there were no effects found in the effluent for the highest exposure concentration and luminescent bacteria EC_50_ values could not be ascertained. It indicated that the effluent experienced no observed toxicity to *Vibrio fischeri*. At the same time, differences in response among the testing organisms were found through the toxicity test. Zabrafish larvae has showed the highest sensitivity of the zebrafish embryos and *Vibrio fischeri* from the above data of toxicity reduction in this experiment. The possible reason that can be used to explain this phenomenon is the embryonic membrane, which selectively restricted big molecular compounds to enter into embryos according to other researchers [42,43]. In zebrafish embryos, the three-layered chorion with pore channels (500–700 nm) could work as an efficient barrier against toxic particles into the embryos [44]. The *Vibrio fischeri* has limitations to its enforceability because of the serious influence of physical-chemical parameters on the tested object [45].

## 5. Conclusions

In this paper, the performance of the combined treatment process of landfill leachate was evaluated by physicochemical analysis, as well as a series of bio-toxicity experiments. The following results were found.

The combined treatment process has shown to be effective not only in the reduction of physicochemical parameters, but also in acute toxicity reduction. The overall removal efficiency of COD, NH_4_^+^-N, TN, Ni, and Cr were up to 93.57%, 97.46%, 73.60%, 93.50% and 87.44%, respectively while the content of Cu, Cd, and Zn were totally removed. Toxicity reduction of landfill leachate to zebrafish larvae, zebrafish embryos, and *Vibrio fischeri* were 85.84%, 87.23%, and 81.52%, respectively. Toxicity test indicated that zebrafish larvae was the most sensitive organism for landfill leachate and most toxic substances were removed by coagulation and sedimentation and electrochemical oxidation process.

The experimental results suggest that the biological and physicochemical combined treatment process with the electrochemical oxidation was an efficient alternative treatment method to reduce toxic organic and inorganic pollutants for the treatment of this kind of landfill leachate.

## Figures and Tables

**Table 1:** Experimental concentration settings.

Model Organisms	Raw	Coagulation and Sedimentation	Anaerobic	Electrochemical Oxidation	Aerobic
Zebrafish embryos	0.50%	2.00%	4.00%	5.00%	6.00%
	0.89%	2.73%	4.90%	6.50%	8.13%
	1.58%	3.74%	5.98%	8.41%	10.96%
	2.82%	5.11%	7.34%	10.90%	14.80%
	5.00%	7.00%	7.00%	14.00%	20.00%
Zebrafish larvae	1.40%	1.50%	2.00%	4.00%	6.00%
	1.49%	1.78%	2.52%	4.76%	7.14%
	1.59%	2.12%	3.16%	5.66%	8.49%
	1.69%	2.52%	3.98%	6.73%	10.09%
	1.80%	3.00%	5.00%	8.00%	12.00%

**Table 2:** Details of the competition of the raw landfill leachate.

Parameter (mg/L)	Raw Landfill Leachate
COD_cr_	1400 ± 72.39
NH_4_^+^-N	394 ± 24.17
TP	1.51 ± 0.33
TN	516 ± 20.98
pH	6.55 ± 0.34
Cu	0.026 ± 0.00162
Zn	1.1651 ± 0.00015
Cd	0.0086 ± 0.0008
Cr	0.2468 ± 0.00133
Ni	0.157 ± 0.00089

**Table 3:** Integrated efficiency process parameters (mg/L).

Source	CODcr	NH_4_^+^-N	Cd	Cr	Ni	Zn	Cu
Raw	1400 ± 72.39	394 ± 24.17	0.0086 ± 0.0008	0.2468 ± 0.00133	0.157 ± 0.00089	1.1651 ± 0.00015	0.026 ± 0.00162
Sample A	607 ± 30.76	340 ± 36.88	0.0015 ± 0.00033	0.077 ± 0.00769	0.15 ± 0.00261	0.0196 ± 0.00046	-
Sample B	571 ± 16.85	315 ± 30	-	0.053 ± 0.00141	0.072 ± 0.00522	0.002 ± 0.00089	-
Sample C	220 ± 31.62	83 ± 5.10	-	0.040 ± 0.00261	0.0136 ± 0.0008	-	-
Sample D	90 ± 3.69	10 ± 1.10	-	0.031 ± 0.00551	0.0102 ± 0.00194	-	-

- Means does not exist. Raw, the raw landfill leachate; sample A, coagulation and sedimentation tank effluent; sample B, anaerobic tank effluent; sample C, electrochemical oxidation tank effluent; and sample D, aerobic tank effluent.

**Table 4:** Seventy-two hour hatching rate and malformation rate of zebrafish embryos exposed to the raw landfill leachate and effluent from four treatment processes.

Toxicity Test	Sample	Parameter	Value (%)	95% CI	TU
Zebrafish embryos	Raw	72 h hatching rate (Vol. %)	1.12	0.85–1.56	89.3
	A		3.66	0.26–7.29	27.3
	B		5.25	5.22–5.68	19.0
	C		7.49	6.59–8.46	13.4
	D		8.95	7.22–11.14	11.2
Zebrafish embryos	Raw	72 h malformation rate (Vol. %)	1.33	0.92–1.85	75.2
	A		4.68	3.95–5.12	21.4
	B		5.41	5.08–5.75	18.5
	C		-	-	-
	D		-	-	-

- Means does not exist. Raw, the raw landfill leachate; sample A, coagulation and sedimentation tank effluent; sample B, anaerobic tank effluent; sample C, electrochemical oxidation tank effluent; and sample D, aerobic tank effluent.
